# Uncovering the role of mitochondrial genome in pathogenicity and drug resistance in pathogenic fungi

**DOI:** 10.3389/fcimb.2025.1576485

**Published:** 2025-04-16

**Authors:** Yue Ni, Xindi Gao

**Affiliations:** ^1^ College of Life and Health Sciences, Northeastern University, Shenyang, Liaoning, China; ^2^ Department of Emergency, Xinqiao Hospital, Army Medical University, Chongqing, China

**Keywords:** mitochondrial genome, *Cryptococcus neoformans*, *Candida albicans*, pathogenicity, drug resistance

## Abstract

Fungal infections are becoming more prevalent globally, particularly affecting immunocompromised populations, such as people living with HIV, organ transplant recipients and those on immunomodulatory therapy. Globally, approximately 6.55 million people are affected by invasive fungal infections annually, leading to serious health consequences and death. Mitochondria are membrane-bound organelles found in almost all eukaryotic cells and play an important role in cellular metabolism and energy production, including pathogenic fungi. These organelles possess their own genome, the mitochondrial genome, which is usually circular and encodes proteins essential for energy production. Variation and evolutionary adaptation within and between species’ mitochondrial genomes can affect mitochondrial function, and consequently cellular energy production and metabolic activity, which may contribute to pathogenicity and drug resistance in certain fungal species. This review explores the link between the mitochondrial genome and mechanisms of fungal pathogenicity and drug resistance, with a particular focus on *Cryptococcus neoformans* and *Candida albicans*. These insights deepen our understanding of fungal biology and may provide new avenues for developing innovative therapeutic strategies.

## Introduction

The rising incidence of fungal infections, with morbidity and mortality rates rising globally, poses a significant public health challenge (Tu et al., 2021; Fisher et al., 2022; Roe, 2023; Denning, 2024). These infections are not only causing substantial health losses but also imposing a considerable financial burden. In the United States alone, direct medical costs are estimated to reach billions of dollars (Koffi et al., 2021; Iliev et al., 2024). The widespread use of antifungal agents has led to the emergence of drug resistance, which has now become a critical public health concern. This resistance not only limits treatment options for patients but also increases the risk of recurrent infections and mortality (Kontoyiannis and Lewis, 2002; Lockhart et al., 2023; Vitiello et al., 2023; Zhang et al., 2023). The World Health Organization (WHO) has released its first Fungal Priority Pathogen List (FPPL), focusing on the most threatening global fungal threats. This has been a major concern for the scientific community, calling on the global research community to strengthen research on fungal infections and drug resistance (Fisher and Denning, 2023; Casalini et al., 2024).

Mitochondria are important organelles in eukaryotic cells, with the role of ATP production and participation in various cellular processes (Singh, 2021; Ziegler et al., 2021; Borcherding and Brestoff, 2023; Ryu et al., 2024). Mitochondria possess their own genomes, termed the mitochondrial genomes, which enable them to synthesize a subset of proteins independently of the nucleus (Sandor et al., 2018; Fu et al., 2020). These proteins play an essential role in cellular energy metabolism, the respiratory chain, and various essential biochemical processes (Wallace, 2016; Kim et al., 2022; Liu et al., 2022; Vercellino and Sazanov, 2022). As eukaryotes evolved, the mitochondrial genome appeared to vary considerably among the major eukaryotic taxa (Zardoya, 2020). In animals, the mitochondrial genomes are typically closed-loop DNA molecules encoding a small number of genes involved in energy production (Boore, 1999). Animal mitochondrial genomes generally lack intergenic regions and introns, rendering them relatively simple (Miyazawa et al., 2021; Pimentel et al., 2024). However, exceptions have been reported in some metazoans with regenerative abilities, such as placozoans and bryozoans (Jenkins et al., 2022). In contrast, the mitochondrial genome of plants is primarily in the form of closed circular DNA molecules containing intergenic regions and varying numbers of introns (Kan et al., 2020; Grosser et al., 2023). Fungal mitochondrial genomes are more similar to plant mitochondria than to animal mitochondria in terms of structure and composition (Sandor et al., 2018). For example, *Saccharomyces cerevisiae* is the first fungus reported to have a cyclic mitochondrial genome and has been extensively studied (Lipinski et al., 2010). Research related to the role of the mitochondrial genome in human fungal pathogens is also on the rise. In pathogenic fungi, the functionality of mitochondria directly influences their physiological status and pathogenic potential (Shingu-Vazquez and Traven, 2011; Gao et al., 2022; Alves and Gourlay, 2024). Mitochondrial proteins are critical for establishing infection through energy metabolism and efficient oxidative stress responses (Bambach et al., 2009; Verma et al., 2018; Soggiu et al., 2019; Zhai et al., 2021). There is also a link between mitochondria and the resistance to antibiotic drugs in pathogenic fungi (Shingu-Vazquez and Traven, 2011; Li et al., 2020; Song et al., 2020; Zhang et al., 2021). The introns of the mitochondrial genome, the stability of the genome, and the generation of reactive oxygen species (ROS) as byproducts can all influence the ability of pathogenic fungi to infect hosts and resist antibiotic drugs (Yildiz and Ozkilinc, 2020; Liu and Pyle, 2021, 2024). In this review, we will focus on outlining the composition and diversity of the mitochondrial genome of pathogenic fungi, the relationship between fungal mitochondrial genome and pathogenicity, and the impact of fungal mitochondrial genome on their drug resistance, with a particular focus on the human opportunistic pathogens *Cryptococcus neoformans* and *Candida albicans.* This review provides valuable information for future studies of fungal mitochondrial systems as well as innovative new therapeutic strategies.

## Mitochondrial genome structure and diversity in pathogenic fungi

Fungal mitochondrial genomes typically exhibit a single circularly mapped chromosome, near-standard bacterial-like tRNA and rRNA structures, and a minimal genetic code (Fonseca et al., 2021; Tang et al., 2024). These mitochondrial genomes encode essential genes required for oxidative phosphorylation and other mitochondrial functions (Lavin et al., 2008; Sandor et al., 2018). Variations in core gene count and organization, along with intergenic region sequences, considerably affect the size of fungal mitochondrial genomes (Aguileta et al., 2014; Franco et al., 2017; Korovesi et al., 2018; de Almeida et al., 2021; Tang et al., 2024). Animal mitochondrial genomes are relatively small, ranging from 14 kb to 20 kb, while plant mitochondrial genomes are larger, ranging from 180 kb to 600 kb (Boore, 1999; Morley and Nielsen, 2017). Fungal mitochondrial genomes fall between these two size ranges, varying from 11,223 bp in *Hanseniaspora pseudoguilliermondii* to 332,165 bp in *Golovinomyces cichoracearum* (Zaccaron and Stergiopoulos, 2021; Christinaki et al., 2022). Most proteins encoded by fungal mitochondrial genomes are key components of oxidative phosphorylation. Nevertheless, there are obvious differences in the amount of these proteins at the species level. For example, seven proteins are encoded in the mitochondrial genome of the model yeast *S. cerevisiae* (Foury et al., 1998; De Chiara et al., 2020) or the related human pathogen *Candida glabrata* (Koszul et al., 2003). However, the mitochondrial genomes of the pathogens *Cryptococcus deneoformans* and *Cryptococcus deuterogattii* encode 13 proteins (Ma and May, 2010), and the mitochondrial genomes of *Aspergillus* and *Penicillium* encode 14 proteins (Joardar et al., 2012). Introns are the main drivers of size variations in fungal mitochondrial genomes. They are widely present in these genomes and are generally classified into Group I and Group II based on RNA secondary structure (Saldanha et al., 1993; Gomes et al., 2023). Variation in intron content is observed even within the same fungal genus. The number of introns, including the *COX1*, *COX2*, *COX3*, *NAD1* and *NAD5* genes, varies considerably between species and occasionally within a single species. Notably, the *COX1* gene is an important reservoir for introns (Ye et al., 2020; Tan et al., 2022), known as a key place where introns are stored. The heterogeneity of mitochondrial introns is closely linked to species evolution. In genera like *Aspergillus* and *Penicillium*, intron addition and deletion occur in a cyclic manner (Nielsen et al., 2004; Joardar et al., 2012). In the *Cordyceps militaris* strain, each intron in the mitochondrial genome may have experienced one to four gain and/or loss events (Zhang et al., 2015). Research indicates that, although the large subunit of ribosomal RNA (LSU rRNA) genes in the majority of fungal species contain highly conserved introns, certain *Candida* species do not possess these introns. This observation implies a correlation between the presence of introns and evolutionary development, emphasizing important variability both among different species and within individual species (Miletti and Leibowitz, 2000; Krol et al., 2019). A recent study used an RNA structure-based bioinformatics approach to identify Group I introns in key mitochondrial genes of pathogenic fungi, including all high-priority pathogens identified by the World Health Organization. The study also assessed the prevalence of these introns in different fungal phylogenies and their fixation within a few genetic hotspots (Liu and Pyle, 2024). It was also found that in *C. albicans* and *Candida auris*, the splicing catalysis of these introns demonstrated greater efficiency compared to previously identified group I introns. Also, these introns exhibited rapid catalytic turnover under ambient temperatures and physiological concentrations of magnesium ions (Liu and Pyle, 2024). This finding implies a potential close relationship between introns and the environmental adaptability of pathogenic fungi.

The human pathogenic fungus *C. neoformans* possesses a mitochondrial genome that ranges in size from 24,740 to 31,327 base pairs (Wang and Xu, 2020). The mitochondrial genome contains 17 genes, exhibiting a conserved gene order across closely related species, such as *C. deneoformans* and *Cryptococcus gattii* (Litter et al., 2005; Ma and May, 2010). This genome encompasses genes responsible for proteins involved in the electron transport chain, ATP synthesis, and genes encoding the mitochondrial ribosomal protein S3 (Rps3) and the small and large subunits of mitochondrial ribosomal RNA (Wang and Xu, 2020). Comparative genomics has revealed that a 40 kb region containing 14 genes was transferred from var. *grubii* to var. *neoformans* (Kavanaugh et al., 2006). These two varieties diverged approximately 18 million years ago (MYA) and share 85-90% nucleotide identity at the genomic level. This nearly identical region is prevalent in most clinical and environmental var. *neoformans* strains globally and the result of a non-reciprocal transfer event from var. *grubii* to var. *neoformans* about 2 MYA. This genetic exchange likely occurred through a hybrid intermediate, potentially formed by incomplete sexual cycling between the variants (Kavanaugh et al., 2006). A study by Yue Wang et al. analyzed 184 sequenced mitochondrial genomes from *C. neoformans* isolates and revealed variations in size, intron distribution, and single nucleotide polymorphisms in the mitochondrial genomes of *Cryptococcus* complex (Wang and Xu, 2020). This genetic diversity is likely associated with the fungal geographic distribution, ecological niche, mating type, and genetic lineage. These variability traits of mitochondrial genomes provide important sights into the genetic diversity and evolution of *Cryptococcus* (Wang and Xu, 2020). The mitochondrial genome of *C. neoformans* shows evidence of frequent intron gains and losses throughout its evolutionary history, as indicated by the distribution and phylogenies of introns and their corresponding exons. The occurrence of self-scissoring introns within mitochondrial genes differs across various complexes and genotypes, presumably as a result of recurrent intron loss and gain events during the homing mechanism (Gomes et al., 2023). In the mitochondrial genomes of *C. neoformans* and *C. gattii*, Group I introns are primarily distributed in the *COB* and *COX1* genes, with varying frequencies among different genotypes and species. For example, the VNIII genotype, a cross between *C. neoformans* and *C. deneoformans*, exhibits the highest intron frequencies in four loci of mtLSU, two loci of *COB*, and five loci of *COX1*. In contrast, lower intron frequencies were observed in the more virulent and frequently isolated VNI genotype in clinical samples worldwide (Gomes et al., 2023). Phylogenetic analyses of *COB* and *COX1* introns reveal that introns occupying the same insertion site form well-defined monophyletic groups. Introns located at the first insertion site in *COB* exhibit greater similarities to one another, whereas those at the second insertion site reveal a similar pattern (Gomes et al., 2023). A survey of Group I introns in the mitochondrial LSU rRNA genes of 77 C*. neoformans* and *C. gattii* isolates identified two new introns in the LSU rRNA genes. These introns form a monophyletic group closely related to the *COX1* introns of certain ascomycete and basidiomycete genera. The structures and sequences of these introns differ from known introns, and their presence is highly associated with pathogenicity and antifungal resistance, suggesting a unique evolutionary history (Gomes et al., 2018).

*C. albicans* is a common commensal and opportunistic pathogenic fungus, and its mitochondrial DNA molecule is 41 kb in size (Jones et al., 2004). This dimorphic fungus is petite-negative, meaning that the absence of mitochondrial DNA renders it non-viable (Bulder, 1964; Chen and Clark-Walker, 2000; Joers et al., 2007). Its mitochondrial genome contains genes encoding complex I subunits that have been lost in closely related fungi, such as in *S. cerevisiae* and *Saccharomyces pombe* (Nosek and Fukuhara, 1994; Anderson et al., 2001). The mitochondrial genome of *C. albicans* has a GC content of approximately 32%, a characteristic shared by many other yeasts (Kolondra et al., 2015), suggesting that it has been conserved to some extent during evolution. Within this mitochondrial genome, GC-enriched fragments are typically dispersed as clusters of short palindromic sequences that may adopt hairpin structures, serving as mobile genetic elements and regulatory signals (Kolondra et al., 2015). The mitochondrial genome of *C. albicans* primarily exists as multiple head-to-tail tandems and includes 14 genes for subunits of the oxidative phosphorylation pathway, two ribosomal RNA genes, and 24 tRNA genes (Gerhold et al., 2010; Kolondra et al., 2015). The protein-coding genes encompass subunits of NADH dehydrogenase (Complex I), cytochrome c oxidase (Complex IV), and ATP synthase (Complex V). The mitochondrial genome has a limited number of introns, specifically two in the *RNL* gene, two in the *COB* gene, and four in the *COX1* gene (Kolondra et al., 2015). The genome consists of two main coding regions: the short coding region (SCR) and the long coding region (LCR), along with two inverted repeat regions (IRa and IRb). These non-coding regions exhibit limited transcriptional activity in RNA sequencing, confined to a few short segments (Kolondra et al., 2015). In *C. albicans*, mitochondrial intergenic regions are useful for examining microvariation (Bartelli et al., 2013). A study conducted the sequencing of mitochondrial genomes from two clinical isolates of *C. albicans*, revealing 372 polymorphic sites. Of these, 230 were located within coding regions and 142 within non-coding regions, indicating a notable presence of neutral substitutions. The high variability and size differences in these non-coding regions, with up to a 56 bp size difference, indicate that they may be subject to neutral evolution (Bartelli et al., 2013). Another study sequenced seven regions of the mitochondrial genomes from 36 C*. albicans* strains and identified 66 polymorphic sites, resulting in the construction of 19 distinct haplotypes. Notably, strains sharing the same mitochondrial haplotypes were found across various geographic regions. The absence of a strong correlation between mitochondrial haplotypes and the origin of strains, whether geographic or anatomical, indicates considerable mobility among various populations (Jacobsen et al., 2008). These findings suggest that the distribution of mitochondrial haplotypes in *C. albicans* is probably independent of geographic distribution or host specificity. Therefore, caution is advised when employing mitochondrial haplotypes in phylogenetic studies of *C. albicans*. A study of mitochondrial genomes from sequenced *C. albicans* isolates representing different sites of infection and countries of origin showed that, in addition to the nuclear genome, the mitochondrial genome also undergoes genetic recombination. This indicates that the mitochondrial genome of *C. albicans* exhibits variability, influenced by sexual or parasexual mating processes (Wang et al., 2018).

## Mitochondrial genome and pathogenic mechanisms

Human fungal pathogens are responsible for severe, life-threatening diseases and predominantly exhibit aerobic characteristics. Therefore, the morphology, genetics, and metabolism of their mitochondria are essential for their survival in environmental contexts and during infections in hosts (Alspaugh, 2015; Chang and Doering, 2018; Song et al., 2020; Rokas, 2022; Thambugala et al., 2024). Mitochondrial dynamics, including their fusion and fission, can impact fungal virulence (Chang and Doering, 2018; Liang et al., 2018). For instance, in *C. albicans*, loss of FZO1, which is involved in mitochondrial fusion, leads to increased susceptibility to peroxide stress and altered drug susceptibility (Thomas et al., 2013). Similarly, in *Aspergillus fumigatus*, a conditional mutant strain of the essential gene Mgm1, encoding another GTPase required for mitochondrial fusion, was completely devoid of virulence (Neubauer et al., 2015). Thus, understanding the functional diversity of fungal mitochondria can provide valuable insights into their biology and pathogenic mechanisms, potentially leading to more effective treatments for fungal infections.

**Figure 1:**
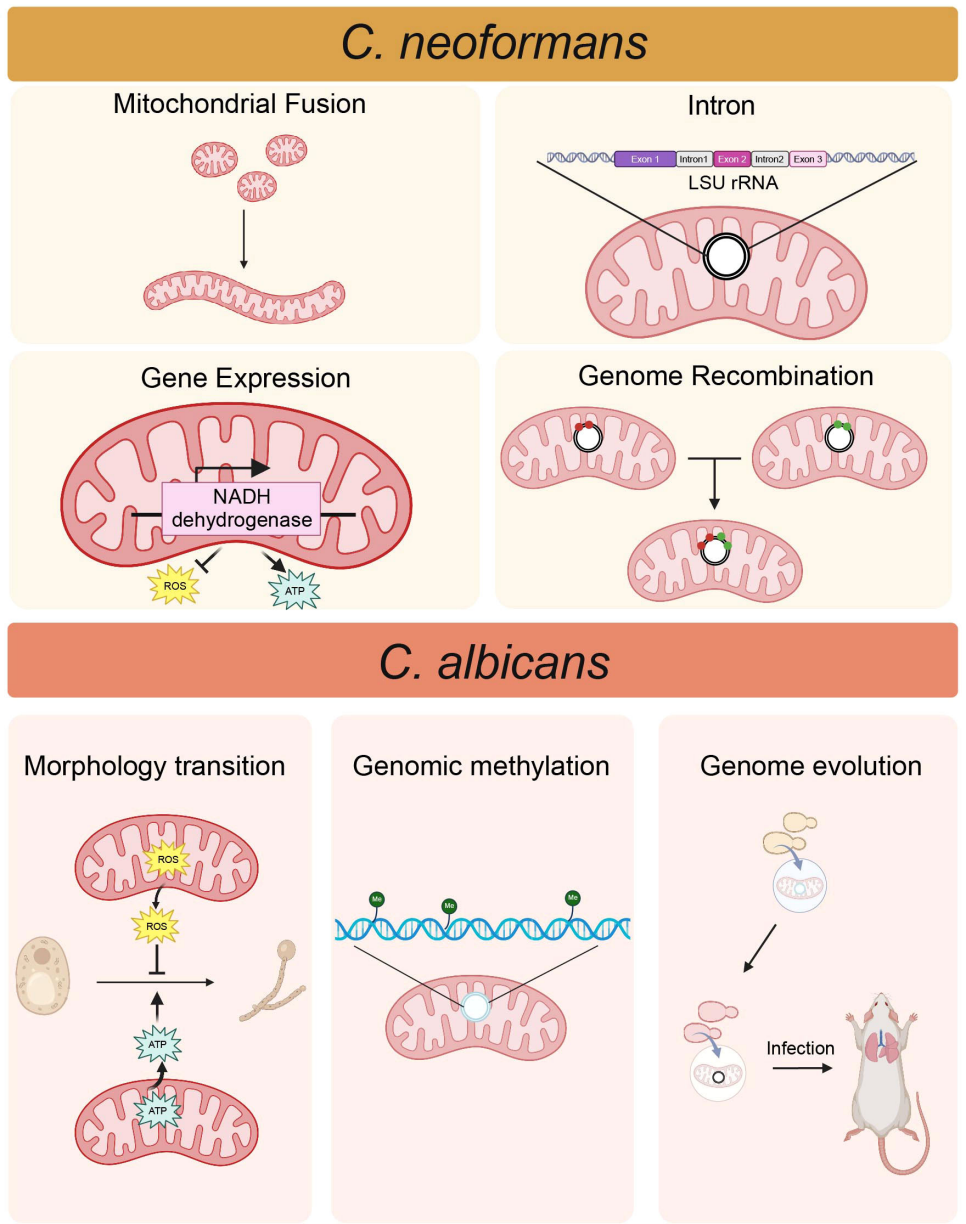
The correlation between mitochondria and the pathogenicity of fungi. In *C. neoformans*, the process of mitochondrial fusion is associated with pathogenicity. Elevated expression of the mitochondrial genome-encoded NADH dehydrogenase gene increases ATP levels while reducing ROS level, thereby enhancing virulence. Mitochondrial genome recombination may facilitate the emergence of more virulent strains. The presence of two novel introns in the mitochondrial LSU rRNA gene correlates with attenuated pathogenicity. In *C. albicans*, the yeast-to-hypha transition necessitates high ATP level, and ROS can inhibit this process. Mitochondrial genome evolution may contribute to the important consequences for host adaption. Mitochondrial genome methylation, recognized as a new epigenetic mechanism, can drive adaptive changes in the mitochondrial genome. (Created with BioRender.com).

The mitochondrial function of *C. neoformans* is directly related to the pathogenicity (**Figure 1**). Specifically, the disruption of mitochondrial fusion attenuates the resistance to oxidative and nitrosative stresses and the virulence of *C. neoformans* (Chang and Doering, 2018). The correlation between the mitochondrial genomes of pathogenic fungi and their virulence is complex. Abnormal expression of mitochondrial genome genes can affect the pathogenicity of *Cryptococcus*. The increased atypical expression of the mitochondrial genome-encoded NADH dehydrogenase gene, caused by a mutation of the NADH dehydrogenase subunit 1 promoter, leads to heightened ATP production, accelerated metabolic activity, and enhanced virulence in *Cryptococcus* (Merryman et al., 2020). Besides, the recombination of mitochondrial genomes may affect the virulence of fungal populations. In *Cryptococcus* species, there is a recombination event of mitochondrial genome in every VNIa-5 isolate, which is associated with disease in HIV-uninfected patients, but not in every non-VNIa-5 isolate, indicating mitochondrial genome recombination may contribute to the emergence of more virulent strains (Ashton et al., 2019). Recent findings indicate that homing endonuclease genes contribute to variations in intron sizes within the mitochondrial LSU rRNA gene of *Cryptococcus* species. These differences may play a role in mitochondrial functionality, potentially affecting virulence (Gomes et al., 2018). The study by Gomes et al. identified two novel introns in the mitochondrial LSU rRNA gene of *C. neoformans*. Notably, the presence of these introns was statistically associated with genotypes reported to be less pathogenic, indicating a potential link between intron presence and reduced virulence (Gomes et al., 2018). Another study revealed evidence of frequent gains and losses of mitochondrial introns during the evolution of *C. neoformans* (Gomes et al., 2023). This indicates a dynamics evolutionary process that could be associated with the fungal adaptation to various host environments and its potential for pathogenicity. Introns within mitochondrial genes can influence the expression of proteins essential for energy production and the response to oxidative stress, both of which are essential for the survival of *C. neoformans* in hostile environments (Fox, 2012; Chang and Doering, 2018; Gomes et al., 2023). The mitochondrial genome is involved in the production of ROS and the ability to manage excessive ROS is also critical for *C. neoformans* to survive the hostile environment (Meng and Ding, 2023). The overexpression of the protein encoded by the mitochondrial genome notably enhanced resistance to increased levels of ROS induced by heat stress, enabling *C. neoformans* to endure high temperatures during host infection (Gao et al., 2022). Nevertheless, the complex relationship between the mitochondrial genome and the virulence of *Cryptococcus* remains largely known and requires further investigation.

In *C. albicans*, genetic diversity within the mitochondrial genomes can have a substantial impact on its pathogenicity by altering the efficiency of energy metabolism, morphological transitions, and responses to oxidative stress (Noble et al., 2010; Bonhomme et al., 2011; Brown et al., 2014; Dantas Ada et al., 2015; Grahl et al., 2015) (**Figure 1**). Energy metabolism plays a critical role during the transition from yeast to hyphae, a process that requires substantial ATP levels and is essential for tissue invasion and damage within the host (Alonso-Monge et al., 2024). It has been reported that reduced respiratory capacity due to mutations in the mitochondrial complex of *C. albicans* attenuates its fitness and virulence (Sun et al., 2019). Moreover, mitochondrial mutants of *C. albicans* exhibited reduced virulence in a mouse model of disseminated disease, while the virulence of all respiratory mutants was similarly diminished in the *Galleria mellonella* infection model (Bambach et al., 2009; Becker et al., 2010; She et al., 2015; Sun et al., 2019). Biofilm is the key virulence factor for *C. albicans* and linked to increased resistance to oxidative stress due to the production of ROS, with mitochondria being the primary source of ROS in eukaryotic cells (Seneviratne et al., 2008; Gulati and Nobile, 2016; Pemmaraju et al., 2016), which indicates a link between mitochondrion and biofilm formation. In addition, mitochondria function as the antioxidant defense system, safeguarding the cell from oxidative damage due to immune system in *C. albicans*. In *C. albicans* strains exhibiting compromised mitochondrial function, there is a decrease in the copy number of the mitochondrial genome, accompanied by an increase in ROS levels. This abnormal ROS level compromises hyphal formation and alters interaction with macrophages, thereby reducing the virulence of the infection (Liang et al., 2018). Genome evolution in *C. albicans*, including changes in the mitochondrial genome such as the accumulation of point mutations, loss of heterozygosity (LOH) events, large-scale chromosomal rearrangements, and even ploidy change, may enhance its ability to survive and reproduce in different host environments (Selmecki et al., 2006; Wu et al., 2007; Ford et al., 2015; Bartelli et al., 2018; Ene et al., 2019). Mitochondrial genome methylation in *C. albicans* has been identified as a novel epigenetic mechanism for the adaptive changes in the mitochondrial genome (Bartelli et al., 2018). Researchers have observed that environmental conditions, such as continuous exposure to hypoxia and 37°C, can decrease mitochondrial genome methylation in strains SC5314 and L757 (Bartelli et al., 2018). This process is closely related to the pathogenicity of *Candida*, as strains that are able to grow and reproduce rapidly are more likely to cause infections. This decreased methylation varies across strains at specific genome locations (Bartelli et al., 2018), suggesting that this response may be lineage-specific and related to the adaptation and different pathogenicity of these strains. However, more direct evidence is needed to further validate whether the altered methylation levels of these loci can directly affect the pathogenicity and environmental adaptability of *C. albicans*.

## Mitochondrial genome and drug resistance

Fungal drug resistance presents a serious and growing challenge in the medical field (Fisher et al., 2022). This resistance is a complex phenomenon influenced by multiple mechanisms that enable fungi to endure and flourish in the presence of antifungal agents. Acquired resistance often involves mutations in target protein binding sites, alterations in drug targets, and the activation of drug efflux mechanisms (Lockhart et al., 2023; Zhang et al., 2023). The mitochondrial function is closely related to the drug resistance in fungi. In *S. cerevisiae*, the lack of the mitochondrial genome results in an obvious upregulation of *PDR5*, a gene that encodes an ATP-binding cassette (ABC) transporter essential for the efflux of antifungal agents, finally enhancing drug resistance (Moye-Rowley, 2005; Rahman et al., 2018; Harris et al., 2021). For pathogenic fungi, such as *C. glabrata*, the loss of the mitochondrial genome can result in increased drug resistance (Sanglard et al., 2001; Batova et al., 2008; Ferrari et al., 2011). In *A. fumigatus*, mitochondrion fission mutant strains exhibit increased resistance to azole drugs (Valero et al., 2020). The loss of mitochondrial genome can also disrupt lipid biosynthesis and subsequently alter membrane permeability, influencing the fungal response to antifungal agents (Calderone et al., 2015; Rella et al., 2016). For *C. neoformans* and *C. albicans*, the loss of mitochondrial function due to tetracycline treatment can increase the susceptibility of *C. neoformans* and *C. albicans* to amphotericin B, a powerful antifungal agent widely used in clinical settings to treat severe fungal infections (Oliver et al., 2008), suggesting the relationship between mitochondrial function and drug resistance varies among different pathogenic fungi.

**Figure 2:**
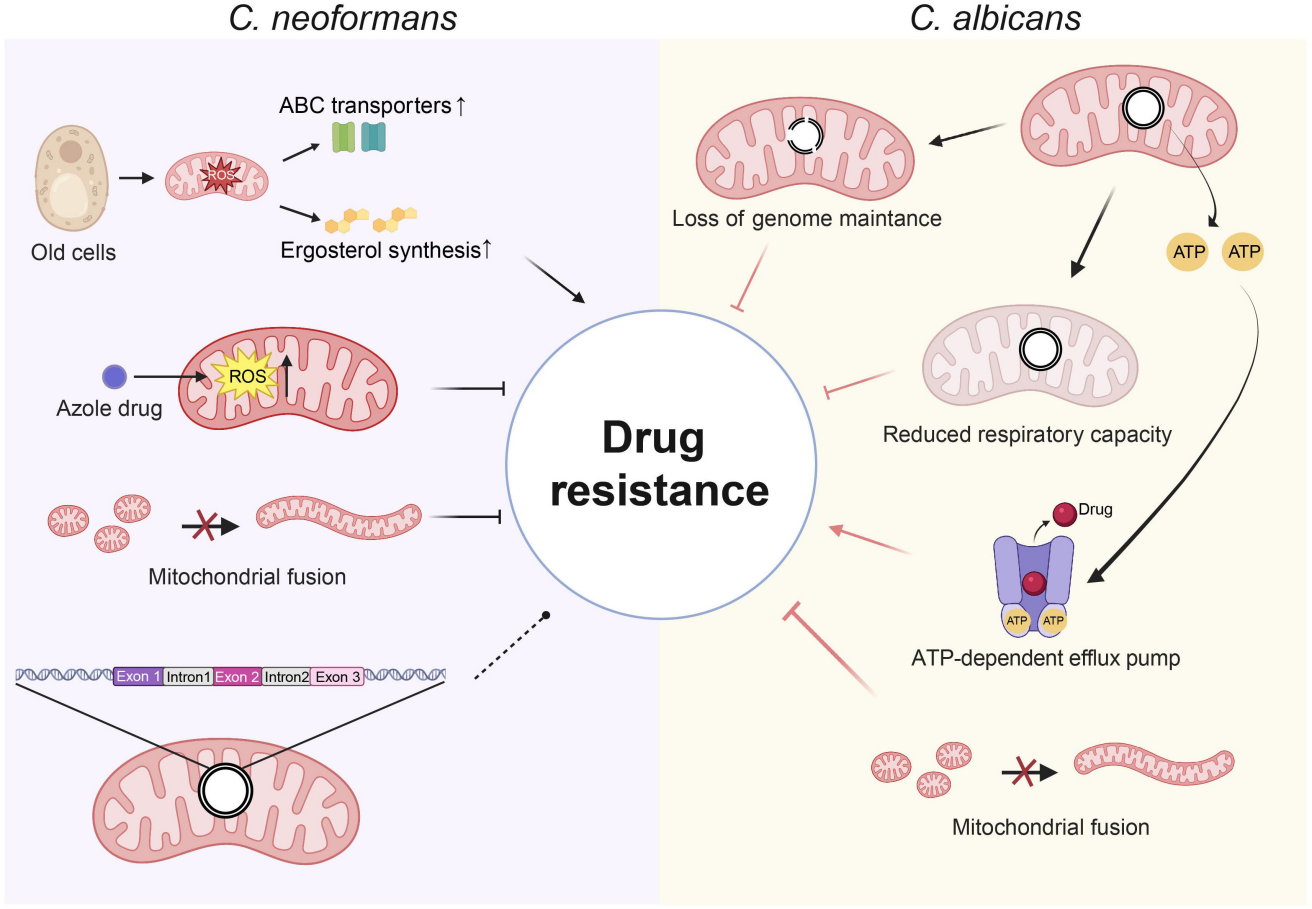
The correlation between mitochondria and antifungal drug resistance. In *C. neoformans*, azoles exert their antifungal effects by increasing intracellular levels of ROS. In old cells, elevated endogenous mitochondrial ROS drive resistance to drugs by increasing ergosterol synthesis and upregulating ABC transporter. The impaired mitochondrial fusion results in reduced resistance to antifungal drugs. Introns in the mitochondrial LSU rRNA gene has an impact on drug susceptibility for amphotericin B and 5-flucytosine. In *C. albicans*, defects in mitochondrial fusion and losses in mitochondrial genome maintenance are associated with diminished resistance to azole drugs. The reduced respiratory capacity diminishes the ability to withstand azole drugs. ATP supply enhances the efficacy of drug efflux pumps, increasing azole efflux and promoting drug resistance. Solid arrows indicate activation, Arrows with a perpendicular bar indicate suppression. Dashed arrows with a circular dot indicate dual effects. (Created with BioRender.com).

*C. neoformans*, like other pathogenic fungi, exhibits drug resistance that is closely associated with mitochondrial functions (**Figure 2**). Research has shown that azoles kill *C. neoformans* by increasing intracellular level of ROS, which are mainly produced by mitochondria (Peng et al., 2018), implying a link between mitochondrion and drug resistance. Impaired mitochondrial fusion in *C. neoformans* results in severe growth defects under hydrogen peroxide stress and is associated with reduced resistance to antifungal agents (Chang and Doering, 2018). Also, a study has pointed out that Cryptococcal mitochondria play a unique role in drug resistance. Age-dependent increases in mitochondrial ROS resulted in regulatory changes in membrane transport proteins and ergosterol synthesis, which enhanced fluconazole tolerance in old *Cryptococcus* cells (Yoo et al., 2024). Thus, ROS have a complex regulatory role in antifungal drug tolerance in *C. neoformans*, with drug-induced ROS inhibiting fungal growth and endogenous ROS in old *C. neoformans* cells enhancing resistance to antifungals. Therefore, further studies are needed to determine whether the differential effects of ROS on drug resistance are due to differences in the types of ROS produced under various conditions. Additionally, dysfunctional mitochondria in *C. neoformans* have been reported to result in reduced mitochondrial membrane potential, increased susceptibility to oxidative stress, while enhanced tolerance to fluconazole (Telzrow et al., 2023), indicating a complex relationship between mitochondrial function and drug resistance. The mitochondrial genome exhibits a profound relationship with antifungal resistance in *C. neoformans*. The investigation focused on group I introns in the mitochondrial LSU rRNA gene and their association with drug susceptibility in 77 *Cryptococcus* isolates, revealing that two novel introns in the LSU rRNA gene may influence the minimum inhibitory concentration of amphotericin B and 5-flucytosine (Gomes et al., 2018). This indicates that group I introns in the mitochondrial genome of *Cryptococcus* may represent valuable molecular markers and therapeutic targets considering antifungal resistance (Gomes et al., 2018). Also, in the process of asexual reproduction, *C. neoformans* passes its mitochondrial genome from parent cells to their progeny. This mechanism can lead to the emergence of new mutations within the mitochondrial genome, especially when subjected to selective pressures like antifungal treatments (Dong et al., 2019; Hua et al., 2019; Wang and Xu, 2020).

In *C. albicans*, deficiencies in mitochondrial fusion and a substantial decline in mitochondrial genome maintenance are linked to diminished resistance to azole antifungals, indicating a connection between the integrity of the mitochondrial genome and drug resistance (Ror and Panwar, 2019), suggesting a relationship between the integrity of the mitochondrial genome and drug resistance (**Figure 2**). The mitochondrial genome is important for respiration and ROS production, both key to developing drug resistance. Mutations in the mitochondrial complex of *C. albicans* lead to reduced respiratory capacity, thereby decreasing its ability to resist azole drugs (Shingu-Vazquez and Traven, 2011; Sun et al., 2013; Thomas et al., 2013; Sun et al., 2019). The function of efflux pumps for antifungal medications requires energy, indicating a possible relationship between the mitochondrial genome and the regulation of drug efflux pump activity (Guo et al., 2014, 2017). In azole-tolerant *C. albicans*, elevated mitochondrial aerobic respiration is associated with an increase in ATP levels, indicating a greater transfer of ATP from the mitochondria to the cytoplasm (Guo et al., 2017). This ensures that drug efflux pumps have a greater supply of available ATP, thereby enhancing intracellular azole efflux. A study has indicated that photodynamic treatment (PDT) presents a promising therapeutic option for *C. albicans*, particularly in light of its multi-drug resistance profile. Notably, the respiratory-deficient strain of *C. albicans* demonstrated an increased susceptibility to PDT compared to its parental strain (Chabrier-Rosello et al., 2008). This finding suggests that the integrity of the mitochondrial genome may play an essential role in the varying responses of *C. albicans* to different therapeutic approaches.

**Figure 3:**
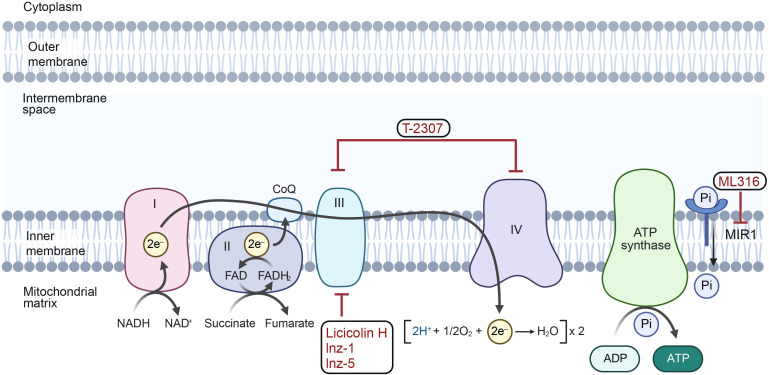
The mechanisms of antifungal agents on mitochondria. T-2307 inhibits the activity of mitochondrial complexes III and IV in the electron transport chain. ML316 suppresses the mitochondrial phosphate carrier Mir1, thus reducing the generation of ATP. Licicolin H, lnz-1 and lnz-5 target mitochondrial respiratory complex III. (Created with BioRender.com).

Although few antifungals currently target the mitochondrial genome directly, several mitochondrion-targeting drugs have shown potential as antifungals (Qin et al., 2023) (**Figure 3**). These agents target unique proteins in the electron transport chain, which are considered promising antifungal targets. For example, T-2307, which inhibits complexes CIII and CIV of the electron transport chain, is in phase II clinical trials, indicating its potential to overcome drug resistance (Shibata et al., 2012; Gerlach et al., 2021). Preclinical agents such as ML316, which targets the mitochondrial phosphate carrier Mir1, exhibit potential in enhancing the effectiveness of current antifungal treatments (McLellan et al., 2018). Licicolin H is a highly effective, broad-range antifungal agent that specifically targets cytochrome bc1 reductase (mitochondrial respiratory complex III). It has shown major inhibitory effects against various species, including *Candida*, *Cryptococcus*, and *Aspergillus* (Singh et al., 2012, 2013). Similarly, indazole compounds Inz-1 and Inz-5 inhibit cytochrome bc1, reducing the mitochondrial respiration of *C. albicans* (Vincent et al., 2016). However, the connection between the mitochondrial genome and drug resistance in pathogenic fungi is not yet fully understood. Therapeutic strategies targeting mitochondrial genome of pathogenic fungi and their encoded proteins will largely have the potential to improve fungal disease treatment, because their genomes and products have characteristics unique to different species.

## Conclusions

### Mitochondrial genome structure and diversity

The mitochondrial genomes of pathogenic fungi display considerable structural and size variability. Typically circular in form, these genomes encode important proteins involved in energy metabolism. Variations in fungal mitochondrial size primarily arise from differences in introns and intergenic regions, along with distinctions in core protein-coding genes. The presence of introns is notable, as they play an important role in contributing to these size differences, thus contributing to genetic diversity within fungal mitochondrial genomes. The dynamics characteristics of these introns, manifested through their acquisition and loss, are intricately linked to the evolutionary processes of species, emphasizing the adaptability of fungal mitochondria.

### Mitochondrial genomes and pathogenicity

The connection between the mitochondrial genome and pathogenicity in fungi is complex. In *C. neoformans*, variations in mitochondrial genome size, mainly due to the presence of introns, are associated with levels of virulence. Strains exhibiting defective tubular mitochondria tend to display reduced virulence characteristics. Besides, the mitochondrial genome plays a critical role in the generation and regulation of ROS, which is essential for the survival of *C. neoformans* within the host. In the case of *C. albicans*, the genetic diversity observed within mitochondrial genomes considerably impacts its pathogenic potential. This impact arises from its influence on the efficiency of energy metabolism, morphological transitions, and responses to oxidative stress. These findings emphasize the essential role that the mitochondrial genome has in fungal pathogenicity.

### Mitochondrial function and drug resistance

The mitochondrial function in pathogenic fungi is key for understanding drug resistance. In *C. neoformans*, the dysfunction of mitochondrion and its byproducts ROS has a complex impact on the resistance to azole drug. In addition, variations in drug susceptibility are linked to introns found in the mitochondrial LSU rRNA gene, indicating that these introns could be used as molecular markers for antifungal resistance. In *C. albicans*, mutations affecting mitochondrial morphology result in reduced resistance against azole drugs. Importantly, the mitochondrial genome plays a role in respiration and ROS production, which is related to drug resistance. However, the direct connection between antifungal drug resistance and the mitochondrial genome remains largely unexplored. More studies are needed to determine whether the mitochondrial genome could be targeted to create better therapeutic strategies for managing fungal infections.
